# DeepBranchAI: a transferable 3D segmentation model for branching networks

**DOI:** 10.3389/frai.2026.1862872

**Published:** 2026-09-16

**Authors:** Alexander V. Maltsev, Lisa M. Hartnell, Luigi Ferrucci

**Affiliations:** Intramural Research Program, National Institute on Aging, Baltimore, MD, United States

**Keywords:** 3D networks, branching networks, clDice, connected components, topology-sensitive segmentation, transfer learning

## Abstract

Three-dimensional branching networks exist throughout biological, natural, and engineered systems as pathways through volumetric space. Segmentation is required to reconstruct these networks for analysis, but minor voxel misclassifications can alter connectivity by falsely disconnecting continuous structures or amplifying spurious branches. Addressing this topological vulnerability requires true 3D segmentation models, since 2D slice-by-slice approaches cannot maintain connectivity across x, y, and z axes. Here, we present DeepBranchAI, a 3D nnU-Net model trained on expert-refined mitochondrial FIB-SEM reference labels as a transferable checkpoint for topology-sensitive segmentation of branching networks. Skeletal-muscle mitochondrial reticulum provides a dense and heterogeneous branching-network training target, and FIB-SEM captures this structure with isotropic nanometer-scale detail. Training on this dataset produced a 3D checkpoint with DSC = 0.942 ± 0.020 across five-fold mitochondrial FIB-SEM validation, providing the basis for subsequent transfer-learning experiments. Transfer learning was evaluated with five-fold cross-validation on paired image/reference-label datasets from 3D-IRCADb venous segmentation, Plant CT roots, and AeroPath airway CT, using continuity-sensitive measures including clDice and absolute connected-component error. Across the external datasets, DeepBranchAI-pretrained fine-tuning had higher mean clDice than scratch nnU-Net. Relative differences in mean clDice were 11.7% for 3D-IRCADb, 5.5% for Plant CT roots, and 1.9% for AeroPath. Mean absolute connected-component error was 25.0% lower for Plant CT roots and 11.7% lower for AeroPath. In 3D-IRCADb, DeepBranchAI-pretrained fine-tuning also had higher mean Dice and clDice than VesselFM, 0.679 versus 0.464 and 0.629 versus 0.360, respectively. The transfer results suggest that training on structurally heterogeneous skeletal-muscle mitochondrial networks can produce 3D features useful beyond the original FIB-SEM domain. Code, trained weights, external-transfer scripts, and validation tables are provided as open-source resources.

## 1 Introduction

Branching structures appear throughout biological, natural, and engineered systems. Examples include airways (Zhang et al., 2023), porous structures (Nair et al., 2020), root systems (Metzner et al., 2015), 3D-printed lattices (Yang et al., 2022), mitochondria in skeletal muscle (Glancy et al., 2015), vasculature in 3D angiographic volumes (Tetteh et al., 2020), and efficient transportation or resource-distribution networks (Banavar et al., 1999). Mapping the connectivity of 3D branching networks is fundamental to understanding their function and integrity, but achieving topologically accurate segmentation is challenging and continues to be an active focus of 3D segmentation research (Shit et al., 2021; Li et al., 2022). Whether naturally occurring or engineered, these networks typically solve a universal challenge: to efficiently distribute resources throughout space while minimizing costs to the system (Banavar et al., 1999; West et al., 1997). The essential first step for measuring relationships in any such system is to determine which components connect and how these relationships are arranged in space. Volumetric imaging requires segmentation, identifying which voxels belong to network structures versus background. Convolutional neural networks (CNNs) provide sophisticated computer-automated support for volumetric segmentation (Ronneberger et al., 2015; Çiçek et al., 2016; Isensee et al., 2021), but branching networks require models that preserve topology as well as voxel-level accuracy (Shit et al., 2021; Li et al., 2022; Clough et al., 2022). Small local errors in complex 3D volumes can disconnect thin branches, merge neighboring structures, or create spurious paths, changing the measured architecture of the network (Shit et al., 2021; Li et al., 2022; Funke et al., 2019).

Several methods and metrics have been developed to address these errors. Some act during neural-network training, while others evaluate whether the final segmentation preserved the network. When a segmentation model is trained, it is adjusted to reduce a loss function, which is a numerical penalty assigned to its mistakes. Topology-aware losses add penalties for broken branches or false merges, rather than judging only whether individual voxels were labeled correctly. In connectomics, maximin affinity learning of image segmentation (MALIS) was introduced for affinity-based neuron segmentation. Instead of only penalizing mislabeled voxels, MALIS penalizes affinity errors that would split one object or merge two objects in the reconstructed network (Funke et al., 2019). Other topology-aware losses use mathematical descriptions of shape to penalize mistakes in connected pieces and loops (Clough et al., 2022). A related strategy is to encode connectivity as a model prior, as in graph-connectivity constrained networks for 3D vessel segmentation (Li et al., 2022).

Connectivity errors have also changed how segmentation performance is measured. Evaluation metrics score the final prediction after training. Segmentation accuracy is often summarized with Dice, a score that measures voxel overlap between the predicted mask and the reference mask. clDice is a related score developed for tubular and branching structures. Instead of measuring only volumetric overlap, clDice compares the skeletonized centerlines of the predicted and reference structures, so missing or disconnected branches are penalized more directly (Shit et al., 2021). These methods improve how segmentation networks are trained and evaluated, but accurate branching-network segmentation also depends on what model is being trained and fine-tuned in the first place.

Reusable 3D segmentation models address this question in related settings. TotalSegmentator is an example of an nnU-Net-trained model that can be applied directly across many CT anatomical structures (Wasserthal et al., 2023). Transfer-learning studies in medical imaging showed that pretrained CNNs can match or outperform models trained from scratch when adapted to new data (Tajbakhsh et al., 2016). Volumetric pretraining extended this idea into 3D, with Med3D co-training 3D models across heterogeneous medical segmentation tasks (Chen et al., 2019) and Models Genesis using self-supervised 3D pretraining to create source models for downstream medical image analysis (Zhou et al., 2021). VesselFM is more directly relevant to branching anatomy because it uses heterogeneous real and synthetic vascular data to build a foundation-model checkpoint for 3D blood-vessel segmentation (Wittmann et al., 2024). These studies show that reusable 3D representations can support segmentation across datasets, motivating a more specific test of whether a checkpoint trained on dense, structurally heterogeneous 3D branching structure can improve fine-tuning on new branching-network datasets.

We imaged mitochondrial networks using focused ion beam scanning electron microscopy (FIB-SEM), which provides nanometer-scale 3D resolution (15 nm isotropic voxels) sufficient to capture the structural details relevant to this study (Xu et al., 2017; Peddie & Collinson, 2014). These mitochondrial networks form dense reticular structures in skeletal muscle and have been mapped previously with volumetric FIB-SEM (Glancy et al., 2015; Caffrey et al., 2019; Vincent et al., 2019). They also exhibit difficulties that recur across 3D branching-network segmentation, including thin interconnections that can be broken apart or falsely amplified by voxel misclassification, variable contrast across imaging volumes, complex branch points, and topological vulnerability (Zhang et al., 2023; Tetteh et al., 2020; Shit et al., 2021; Li et al., 2022; Rudyanto et al., 2014). To generate reference labels for this source domain, we used sparsely trained conventional machine-learning models to bootstrap preliminary mitochondrial segmentations, then manually refined those drafts into an expert-corrected reference-label set for 3D model training. DeepBranchAI is the resulting 3D nnU-Net checkpoint trained on these expert-refined mitochondrial FIB-SEM labels. We evaluate it first in the source domain using five-fold mitochondrial cross-validation and topology-sensitive metrics, then test whether the learned checkpoint can serve as a transferable initialization for other 3D branching-network segmentation tasks.

The three external branching-network datasets with distinct imaging modalities and structures used here were 3D-IRCADb venous-system CT (Soler et al., 2010), Plant CT roots (Harwood, 2024), and AeroPath airway CT (Støverud et al., 2024). DeepBranchAI-pretrained fine-tuning is compared with scratch nnU-Net training and with a domain-relevant comparator for each external dataset, including VesselFM for venous CT, a 3D U-Net root-segmentation baseline informed by prior root-system work, and an airway-specific, cross-modality MurineAirwaySegmentation checkpoint adapted to AeroPath airway CT (Wittmann et al., 2024; Selzner et al., 2023; Isensee et al., 2023). Performance is evaluated with voxel-overlap and continuity-sensitive measures, including Dice, clDice, and absolute connected-component error. DeepBranchAI-pretrained fine-tuning had higher mean clDice than scratch nnU-Net in all three datasets and had lower mean absolute connected-component error in the Plant CT root and AeroPath airway CT tasks.

## 2 Related work

Deep learning has become a standard approach for volumetric biomedical segmentation, building from U-Net and 3D U-Net to the self-configuring nnU-Net framework (Ronneberger et al., 2015; Çiçek et al., 2016; Isensee et al., 2021). This has also enabled reusable 3D segmentation systems and pretrained models. TotalSegmentator is an nnU-Net-trained model developed for out-of-the-box CT segmentation across many anatomical structures (Wasserthal et al., 2023), while Med3D and Models Genesis explored reusable 3D source models through supervised and self-supervised volumetric pretraining (Chen et al., 2019; Zhou et al., 2021). Several models have targeted tubular or branching anatomy, including DeepVesselNet, graph-connectivity constrained networks, VesselFM, VesselNN for multiphoton microscopy vasculature segmentation, and CS2-Net (Tetteh et al., 2020; Li et al., 2022; Wittmann et al., 2024; Teikari et al., 2016; Mou et al., 2021). General architecture work such as TransUNet-style transformer U-Net designs has also influenced biomedical segmentation (Chen et al., 2024), and additional vessel-focused systems include 3DVascNet, D2-RD-UNet, Ori-Net, and coronary-geometric refinement methods (Narotamo et al., 2024; Cavicchioli et al., 2025; Jiang et al., 2024; Jiang et al., 2024). Together, these studies show that the field has moved from isolated segmentation models toward reusable 3D representations and architecture designs for branching structures.

Topology-aware losses and metrics are especially important for branching networks because strong voxel overlap can still hide broken or falsely merged structures. clDice measures centerline overlaps for tubular structures, persistent-homology losses describe connected components and loops, and graph-connectivity constrained networks explicitly encode vascular connectedness (Shit et al., 2021; Li et al., 2022; Clough et al., 2022). Related efforts address the label-generation side of the problem, including cross-model self-correction approaches such as AIDE, human-in-the-loop tools such as MONAI Label and Cellpose 2.0, and GAN-generated TOF-MRA image/label pairs (Wang et al., 2021; Diaz-Pinto et al., 2024; Pachitariu & Stringer, 2022; Subramaniam et al., 2022). Public datasets are also essential for training and evaluating models on continuous volumetric networks. Examples include MitoEM for 3D mitochondrial instance segmentation (Wei et al., 2020), 3D-IRCADb for abdominal CT with expert segmentations used here for venous-system segmentation (Soler et al., 2010), MIDAS TOF-MRA brain MRA data used in cerebrovascular-segmentation studies (Marron, n.d.), VESSEL12 for pulmonary-vessel CT (Rudyanto et al., 2014), and SMILE-UHURA for small-vessel segmentation (Chatterjee et al., 2024). DeepBranchAI builds on this background by evaluating whether a 3D nnU-Net checkpoint trained on expert-refined, structurally heterogeneous mitochondrial FIB-SEM labels can serve as a transferable initialization for continuity-preserving segmentation across independent branching-network domains. The evaluation datasets are summarized in Table 1.

**Table 1:** Datasets used in the DeepBranchAI evaluation.

Dataset	Structure	Branching style	Study role
Skeletal-muscle mitochondrial dataset (FIB-SEM)	Human skeletal-muscle mitochondrial reticulum	Dense intracellular branching network with heterogeneous surrounding ultrastructure	Source-domain training and 5-fold validation
3D-IRCADb venous system (CT) (Soler et al., 2010)	Abdominal venous system	Clinical vascular network with elongated tubular anatomy	External fine-tuning
3D plant-root dataset (CT) (Harwood, 2024)	Plant root systems	Non-animal biological branching network with thin continuous roots	External fine-tuning
AeroPath airway dataset (CT) (Støverud et al., 2024)	Pulmonary airway trees	Clinical airway network with distal branches and pathological variation	External fine-tuning

## 3 Methods

### 3.1 Overview of DeepBranchAI model development

DeepBranchAI was developed through three stages (Figure 1). Stage 1 (Preprocessing and Training Set Curation) prepared a heterogeneous training set from our FIB-SEM image volumes. Stage 2 (Training Set Generation) iteratively built and refined reference labels through a label-generation process, beginning with conventional machine learning for preliminary segmentation and continuing through expert refinement cycles that transitioned to deep learning. Stage 3 trained the DeepBranchAI 3D nnU-Net checkpoint evaluated in the mitochondrial validation and transfer experiments.

**Figure 1:**
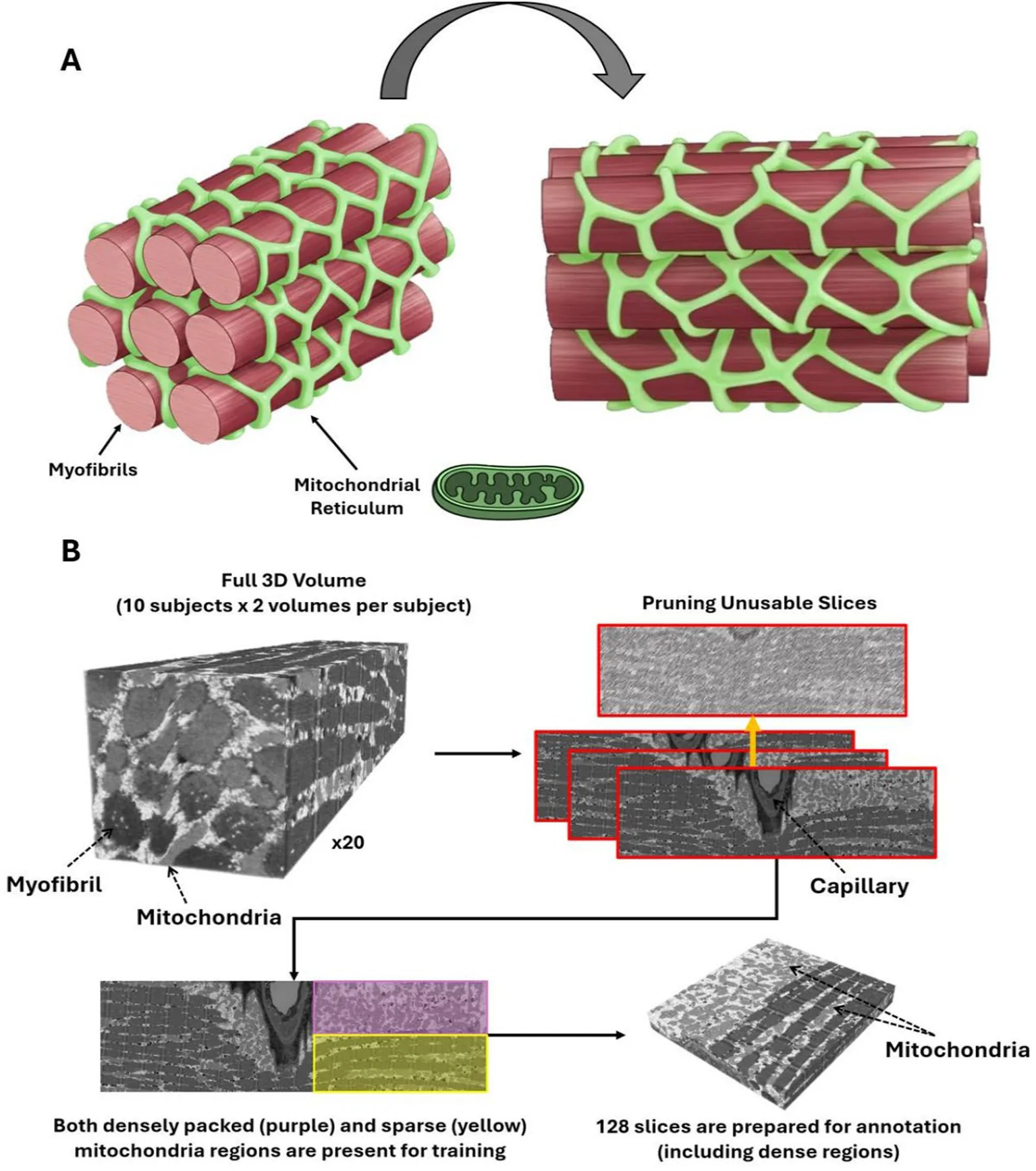
DeepBranchAI model development Stage 1: Volume preparation. **(A)** Schematic showing mitochondria interspersed between myofibrils in human skeletal muscle. Many of them are in contact with each other, forming a nearly continuous branching network. **(B)** Preprocessing includes cross-correlation alignment to ensure 3D continuity and artifact removal to prevent spurious training signals. Optional frequency-domain denoising improves signal-to-noise ratio (SNR) while preserving edges. The training set was curated to capture topological diversity. It is visually evident that densely packed mitochondria near capillaries (purple) are organized differently from regions with more linear, sparse arrangements (yellow). Capturing this diversity supports 3D contextual learning and balanced representation that helps reduce class bias.

### 3.2 Software and environment

Segmentation was performed using the following software environment:

- Python: 3.12.2
- PyTorch: 2.2.1 with CUDA 11.8
- nnU-Net: v2.3.1
- ORS Dragonfly: 4.0 (for manual annotation and refinement, https://dragonfly.comet.tech/)
- ImageJ/Fiji: with Trainable Weka Segmentation plugin (for initial 2D segmentation)
- Python packages: nibabel, tifffile, numpy, multiprocessing
- Destriping: aind-smartspim-destripe package (https://github.com/AllenNeuralDynamics/aind-smartspim-destripe)

### 3.3 Preprocessing and training set curation

We built the training set to be deliberately heterogeneous. It includes image volumes from processed samples of skeletal muscle biopsies from 10 participants. Images were obtained as TIFF stacks using FIB-SEM, then aligned and binned to produce volumes with 15 nm isotropic resolution (Xu et al., 2017; Caffrey et al., 2019). Both images and labels were converted to NIfTI format (.nii.gz) using the Python *nibabel* library, with segmentation labels stored as binary masks.

#### 3.3.1 Preprocessing steps

Raw stacks undergo three preprocessing operations (Figure 1):

1 Cross-correlation and volume alignment correct spatial misalignments between slices, ensuring structures remain continuous throughout the volume rather than identifying false positives or false negatives.
2 Interference slice removal identifies and removes erroneous TIFF slices that disrupt continuity through automated detection of intensity anomalies or manual inspection.
3 Denoising optionally applies wavelet-FFT filtering to remove stripe artifacts common in scanning microscopy (Münch et al., 2009). The filter uses a Daubechies-3 wavelet with two parameter configurations: cellular images (sigma = 64, max_threshold = 3) and non-cellular regions (sigma = 128, max_threshold = 12). Implementation is provided in Demo_Destripe.ipynb in the GitHub repo.

#### 3.3.2 Training set balancing

This curation phase creates a balanced dataset focusing on continuous 3D branching structures (Figure 1):

4 Sub-structure verification ensures that features are correctly identified through expert visual inspection. For branching networks, this means including different topological configurations (clustered vs. distributed patterns, peripheral vs. central locations).
5 Depth requirement establishes a minimum threshold of 128 Z-slices (~10% of the maximum z depth of our volume), providing sufficient volumetric context for learning 3D topology (Figure 1B).
6 Class balancing provides approximately equal representation of structural sub-types by cropping more training patches from underrepresented regions.

### 3.4 Mitochondrial reference label generation

The label-generation process leverages conventional machine learning and deep learning in complementary roles. Conventional ML algorithms achieve reasonable accuracy with limited training data by training with globally effective pre-defined image features, while deep learning requires more initial input but scales better once enough corrected labels exist (Breiman, 2001; Litjens et al., 2017).

#### 3.4.1 Initial conventional ML stage

7 Preliminary segmentation uses the 2D Weka Trainable Segmentation algorithm (Arganda-Carreras et al., 2017) (ImageJ implementation). Training regions are marked by drawing on representative target and background regions. This stage used conventional ML to generalize from sparse labels.
8 Expert refinement corrects obvious errors through manual editing using ORS Dragonfly 4.0. Corrections are saved as overlays and converted to binary masks. Refinement and segmentation were performed in three dimensions because different axes can reveal different branching morphology.
9 Iterative retraining repeats steps 7–8 until reaching saturation, determined through expert validation when visual inspection shows no obvious errors remaining. In this study, 2–3 iterations were sufficient.
10 High-quality extraction identifies the most accurate segments through expert visual inspection. The goal is to identify at least 32 consecutive XY slices with high-quality annotations to begin 2D deep learning training.

#### 3.4.2 Transition to deep learning

11 2D neural network training uses the extracted segments to train a 2D nnU-Net model, marking the transition from conventional ML to deep learning.
12 Inference on full dataset applies the trained 2D network to generate probability maps across the entire dataset. These maps guide experts to focus on ambiguous regions.
13 Final expert refinement uses a 0.50 threshold on probability maps to produce initial binary predictions, which experts further corrected to create reference labels for the 3D neural network.

The full Stage 2 reference-label construction process is summarized in Figure 2.

**Figure 2:**
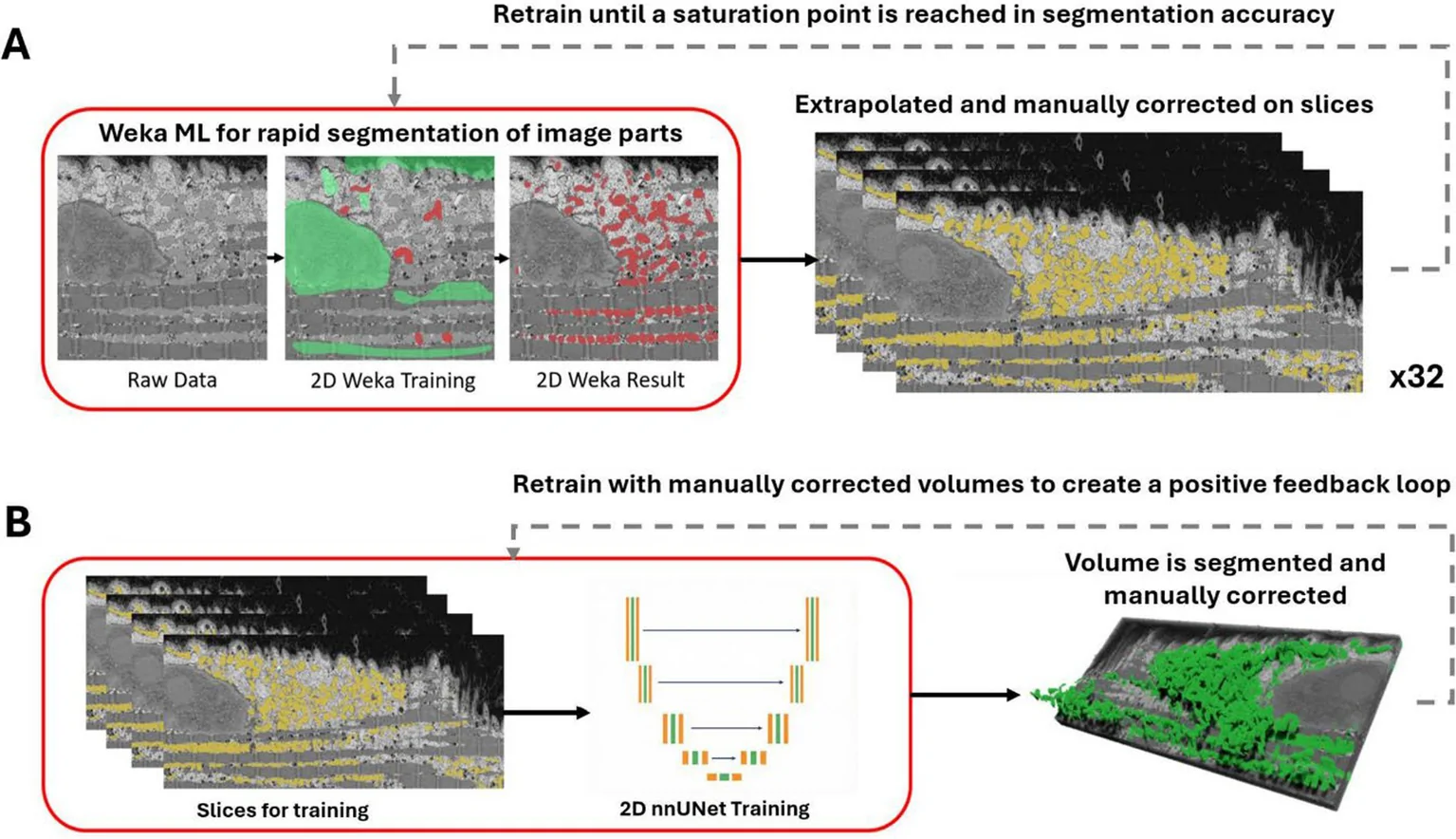
DeepBranchAI model development Stage 2: Iterative reference label Construction. **(A)** The process starts with a 2D Weka random forest classifier trained on minimal annotations. Experts correct the output and retrain iteratively until accuracy plateaus. **(B)** The refined segments are then used to train a 2D nnU-Net, which generates probability maps across full volumes. Experts do a final refinement pass on these maps, producing reference-label volumes that feed back into further training.

### 3.5 Training the DeepBranchAI 3D checkpoint

In Stage 3, the 3D nnU-Net is trained using a chunk size of 352 × 352 × 128 voxels. We refer to the resulting model as DeepBranchAI (Figure 3). This chunk size captures both broad contextual information and fine-grained features necessary for topology preservation.

**Figure 3:**
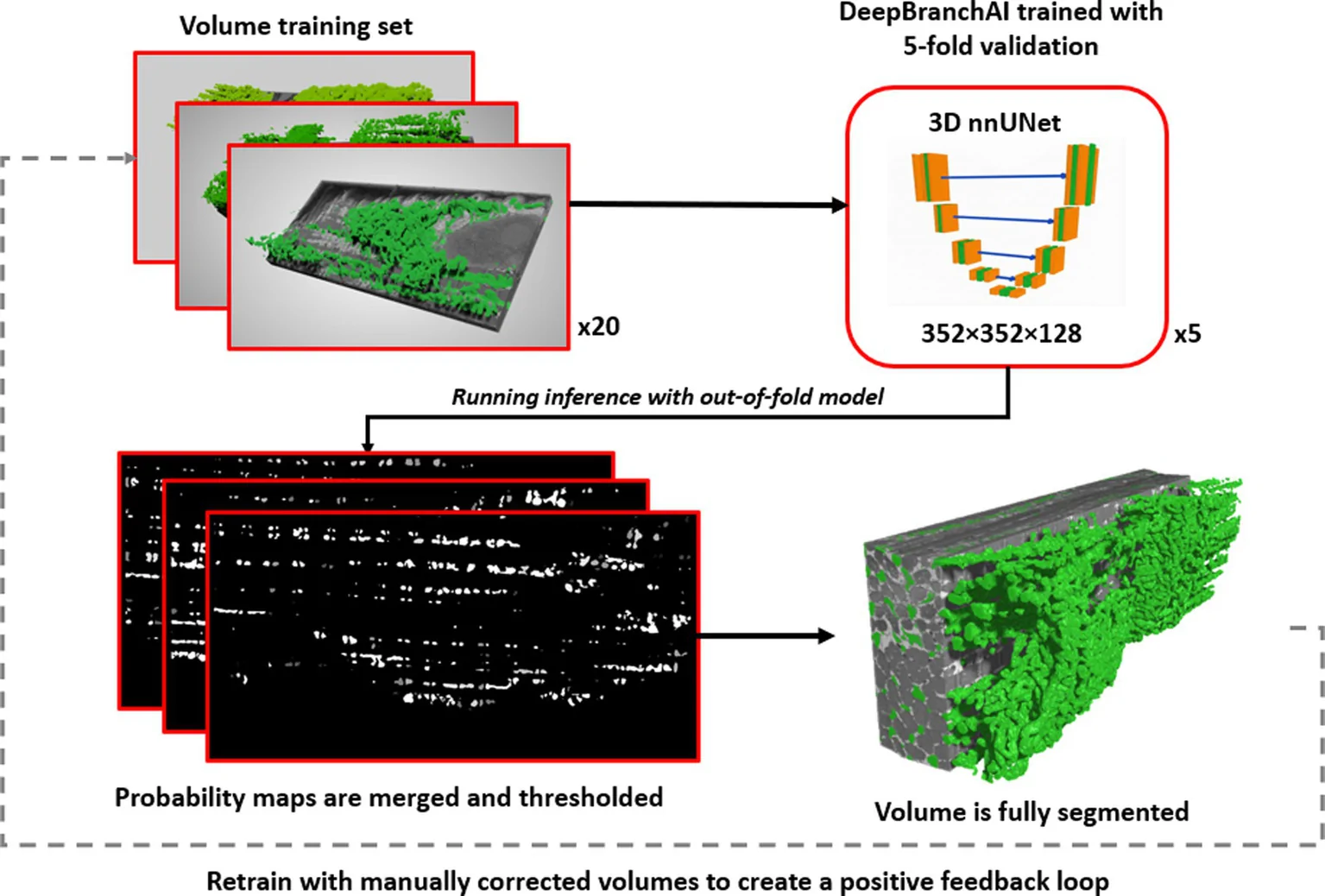
DeepBranchAI model development Stage 3: DeepBranchAI training and inference. The 3D nnU-Net model, referred to here as DeepBranchAI, is trained using high-quality reference labels with chunk sizes large enough to capture local details and long-range topological relationships. Trained models segment new volumes through chunk-wise processing, generating probability maps that are stitched and thresholded to produce final segmentations.

#### 3.5.1 Training and inference

14 3D neural-network training uses the automatic hyperparameter configuration from nnU-Net (Isensee et al., 2021) for 100 epochs with 5-fold cross-validation. The reference-label set contained 20 volumes, with 4 volumes held out for validation in each fold. The reported chunk size was selected for the 48 GB VRAM workstation used here. A 24 GB VRAM workstation would accommodate approximately half of this patch volume, with nnU-Net automatically adapting patch size to available memory.
15 Automated inference applies each fold’s held-out model to that fold’s validation volumes using nnU-Net inference algorithms, which handle chunk-wise processing with overlapping 3D patches. The final segmentation volume is assembled via stitching and weighted averaging.
16 Thresholding applies a 0.50 cutoff to the merged probability map to generate the final binary segmentation.
17 Post-processing for reported metrics used the nnU-Net validation outputs directly after thresholding. No optional connected-component filtering or manual refinement was applied to the predictions used in the quantitative validation tables.

### 3.6 Code availability and implementation resources

The complete codebase is provided through Jupyter notebooks: *Data Preprocessing*

- Demo_Destripe.ipynb: Wavelet-FFT filtering for stripe artifact removal with parallelized processing.
- Training Data Preprocessing.ipynb: Quality control including dimension verification and intensity statistics.

*Model Training and Evaluation*

- nnUNET 3D Train.ipynb: End-to-end training for 3D nnU-Net models
- nnUNET 3D Inference.ipynb: Batch inference for generating 3D segmentation predictions.
- Validate 3D DeepBranchAI.ipynb: Evaluation metrics for 3D models with volumetric analysis.

*Transfer Learning Validation*

- External-transfer scripts: Plant CT root, AeroPath airway CT, and 3D-IRCADb venous-system fine-tuning and metric-generation scripts, including scratch versus DeepBranchAI-pretrained comparisons and topology/continuity metric summaries.

All notebooks and trained model weights are available at https://github.com/alexmaltsev/DeepBranchAI.

### 3.7 Evaluation metrics

We used overlap, agreement, class-balance, and topology-sensitive metrics to assess segmentation performance (Shit et al., 2021; Taha & Hanbury, 2015). The mitochondria reference labels are the expert-refined Stage 2 annotations used for Stage 3 training and validation. Let TP, TN, FP, and FN denote true positives, true negatives, false positives, and false negatives, respectively:

Precision measures the fraction of predicted target voxels that are true target voxels:


$$\text{Precision}=\frac{\mathrm{TP}}{\mathrm{TP}+\mathrm{FP}}$$

Sensitivity (Recall) measures the proportion of actual target voxels correctly identified:


$$\text{Sensitivity}=\frac{\mathrm{TP}}{\mathrm{TP}+\mathrm{FN}}$$

Specificity measures the proportion of background voxels correctly identified:


$$\text{Specificity}=\frac{\mathrm{TN}}{\mathrm{TN}+\mathrm{FP}}$$

Accuracy measures the overall proportion of correctly classified voxels:


$$\text{Accuracy}=\frac{\mathrm{TP}+\mathrm{TN}}{\mathrm{TP}+\mathrm{TN}+\mathrm{FP}+\mathrm{FN}}$$

Dice Similarity Coefficient (DSC) measures overlap between predicted and reference segmentations (Taha & Hanbury, 2015):


$$\mathrm{DSC}=\frac{2\times \mathrm{TP}}{2\times \mathrm{TP}+\mathrm{FP}+\mathrm{FN}}$$

Absolute Volume Difference (AVD) quantifies relative difference in total segmented volume:


$$\mathrm{AVD}=|\frac{V_{\text{pred}}-V_{\mathrm{GT}}}{V_{\mathrm{GT}}}|\times 100\%$$

where $V_{\text{pred}}$ and $V_{\mathrm{GT}}$ represent the predicted and reference volumes, respectively.

Cohen’s Kappa measures agreement while accounting for chance:


$$\kappa =\frac{p_{o}-p_{e}}{1-p_{e}}$$

where $p_{o}$ is observed agreement and $p_{e}$ is expected agreement by chance. Values above 0.8 indicate excellent agreement.

clDice measures branch-continuity agreement between predicted and reference segmentations by comparing their skeletonized centerlines. The clDice formula follows Shit et al. (Shit et al., 2021). Let P be the predicted binary mask, R be the reference binary mask, S(P) be the skeleton of the prediction, and S(R) be the skeleton of the reference:

Topology precision is:


$$T_{\text{prec}}=\frac{|S(P)\cap R|}{|S(P)|}$$

Topology sensitivity is:


$$T_{\text{sens}}=\frac{|S(R)\cap P|}{|S(R)|}$$

clDice is the harmonic mean of topology precision and topology sensitivity:


$$\text{clDice}=\frac{2T_{\text{prec}}T_{\text{sens}}}{T_{\text{prec}}+T_{\text{sens}}}$$

Absolute connected-component error measures absolute component-count discrepancy by comparing the absolute difference between the number of predicted and reference connected objects. Connected components were computed using 26-connectivity. Let $C(P_{i})$ be the number of connected components in the predicted mask for validation case i and let $C(R_{i})$ be the number of connected components in the reference mask for the same case.

Per-case absolute connected-component error is:


$$E_{\mathrm{CC},i}=|C(P_{i})-C(R_{i})|$$

Mean absolute connected-component error across (*N*) validation cases is:


$${\bar{E}}_{\mathrm{CC}}=\frac{1}{N}\sum_{i=1}^{N}|C(P_{i})-C(R_{i})|$$

Summary values in Tables 2–5 are reported as the unweighted mean ± sample standard deviation across folds 0–4. Standard deviations were calculated from the unrounded fold-level values. The primary statistical analysis compared DeepBranchAI-pretrained fine-tuning with scratch nnU-Net using paired out-of-fold values for each held-out volume. Two-sided Wilcoxon signed-rank tests were performed separately for Dice, clDice, and absolute connected-component error in each external dataset. Holm correction was applied across the nine comparisons, with adjusted *p* < 0.05 considered significant. For two Plant CT cases with nonempty reference segmentations but zero predicted centerline overlap, clDice was assigned 0 rather than treated as undefined. All 29 Plant CT volumes were retained.

**Table 2:** Expanded volume-wise 5-fold mitochondrial validation for DeepBranchAI 3D nnU-Net.

Metric	Fold 0	Fold 1	Fold 2	Fold 3	Fold 4	Mean ± SD
Dice	0.956	0.920	0.962	0.920	0.951	0.942 ± 0.020
Precision	0.948	0.988	0.953	0.966	0.956	0.962 ± 0.016
Sensitivity	0.964	0.863	0.972	0.880	0.946	0.925 ± 0.050
Specificity	0.994	0.999	0.995	0.996	0.994	0.996 ± 0.002
clDice	0.944	0.904	0.961	0.948	0.943	0.940 ± 0.021
Abs. CC error	196	186	59	398	232	214 ± 122
AVD (%)	2.644	12.634	1.957	8.841	4.083	6.032 ± 4.565
Kappa	0.951	0.914	0.958	0.910	0.944	0.935 ± 0.022

The final column reports the unweighted mean +/- sample SD across folds 0-4.

**Table 3:** Stage 3 mitochondrial segmentation-model comparison and clDice-loss training.

Metric	2D U-Net	3D U-Net	2D nnU-Net	DeepBranchAI (CE + Dice)	DeepBranchAI (CE + Dice + clDice)
Dice	0.789 ± 0.051	0.907 ± 0.047	0.890 ± 0.046	0.942 ± 0.020	0.941 ± 0.019
Precision	0.796 ± 0.084	0.948 ± 0.012	0.910 ± 0.050	0.962 ± 0.016	0.953 ± 0.017
Sensitivity	0.794 ± 0.035	0.877 ± 0.083	0.874 ± 0.067	0.925 ± 0.050	0.932 ± 0.052
Specificity	0.978 ± 0.011	0.994 ± 0.002	0.990 ± 0.006	0.996 ± 0.002	0.995 ± 0.003
clDice	0.735 ± 0.095	0.879 ± 0.068	0.879 ± 0.041	0.940 ± 0.021	0.941 ± 0.028
Abs. CC error	7,565 ± 3,790	1,267 ± 648	1,373 ± 577	214 ± 122	183 ± 108
AVD (%)	14.554 ± 5.404	9.465 ± 7.949	7.820 ± 3.665	6.032 ± 4.565	6.267 ± 3.456
Kappa	0.766 ± 0.057	0.898 ± 0.051	0.878 ± 0.057	0.935 ± 0.022	0.934 ± 0.021

Values are unweighted mean ± sample SD across folds 0–4.

**Table 4:** Five-fold Z-depth ablation for 352 × 352 × Z patches.

Metric	32 Z-depth	64 Z-depth	128 Z-depth
Precision	0.954 ± 0.020	0.955 ± 0.014	0.962 ± 0.016
Sensitivity	0.917 ± 0.067	0.922 ± 0.057	0.925 ± 0.050
Specificity	0.995 ± 0.003	0.995 ± 0.002	0.996 ± 0.002
Dice	0.933 ± 0.035	0.936 ± 0.025	0.942 ± 0.020
clDice	0.931 ± 0.033	0.936 ± 0.029	0.940 ± 0.021
Abs. CC error	230 ± 226	203 ± 96	214 ± 122
AVD (%)	6.664 ± 5.076	6.718 ± 4.375	6.032 ± 4.565
Kappa	0.926 ± 0.039	0.930 ± 0.027	0.935 ± 0.022

Values are unweighted mean ± sample SD across folds 0–4.

**Table 5:** External transfer validation on paired image/reference-label datasets.

Dataset	Model	Dice (mean ± SD)	clDice (mean ± SD)	Abs. CC error (mean ± SD)
3D-IRCADb venous system (Soler et al., 2010) (*n* = 12)	VesselFM fine-tune (Wittmann et al., 2024)	0.464 ± 0.056	0.360 ± 0.098	111.47 ± 45.91
	Scratch nnU-Net	0.638 ± 0.093	0.563 ± 0.100	25.60 ± 3.24
	DeepBranchAI-pretrained fine-tune	0.679 ± 0.080	0.629 ± 0.100	27.17 ± 5.06
	% difference over best	+6.43%	+11.72%	−6.13%
Plant root CT (Harwood, 2024) (*n* = 29)	3D U-Net root baseline	0.459 ± 0.066	0.403 ± 0.079	97.95 ± 27.80
	Scratch nnU-Net	0.557 ± 0.023	0.568 ± 0.060	25.47 ± 17.05
	DeepBranchAI-pretrained fine-tune	0.611 ± 0.053	0.599 ± 0.062	19.11 ± 13.07
	% difference over best	+9.69%	+5.46%	+24.97%
AeroPath airway CT (Støverud et al., 2024) (*n* = 27)	MurineAirwaySegmentation cross-modality fine-tune (Isensee et al., 2023)	0.831 ± 0.043	0.768 ± 0.031	76.13 ± 18.72
	Scratch nnU-Net	0.879 ± 0.031	0.824 ± 0.019	56.12 ± 14.23
	DeepBranchAI-pretrained fine-tune	0.880 ± 0.033	0.840 ± 0.014	49.57 ± 15.77
	% difference over best	+0.11%	+1.94%	+11.67%

Standard deviations were calculated from the unrounded fold-level values. Percentage rows were calculated from the displayed mean values.

### 3.8 Computational requirements

All procedures were performed on a workstation with an NVIDIA RTX A6000 (48 GB VRAM), 128 GB RAM, and 24 logical processors running Ubuntu 24. Typical training times: approximately 30 min per iteration for 2D Weka segmentation, 4–6 h for 2D nnU-Net training, and 2–3 days per fold for 3D nnU-Net five-fold cross-validation training.

## 4 Results

We evaluate the trained DeepBranchAI 3D checkpoint in five ways. First, validation on the skeletal-muscle mitochondrial FIB-SEM dataset is reported with overlap, class-balance, and topology-sensitive metrics. Second, Stage 3 model comparisons test whether the final 3D nnU-Net checkpoint improves segmentation relative to 2D and standard 3D U-Net alternatives. Third, a controlled clDice-loss ablation tests a topology-aware loss. Fourth, a five-fold Z-depth ablation tests whether reducing the axial context from 128 slices to 64 or 32 slices degrades continuity-sensitive performance. Fifth, external fine-tuning evaluates transfer on paired public image/reference-label datasets.

### 4.1 Quantitative evaluation of DeepBranchAI

DeepBranchAI demonstrated strong performance in segmenting complex branching networks from FIB-SEM volumetric data with fold Dice ranging from 0.920 to 0.962 (Table 2).

#### 4.1.1 Cross-validation performance

We used five-fold cross-validation with an 80/20 train-validation split, grouping volumes by subject so that no subject appeared in both sets within a fold. Metrics were computed volume-wise on the full 128-slice held-out validation volumes after prediction stitching or assembly. Across all 20 held-out volumes, foreground mitochondria represented 10.2% of voxels in the reference labels and background represented 89.8%. Precision was therefore reported alongside sensitivity and specificity to avoid relying on specificity alone in this background-dominant setting. Across the five folds, the unweighted mean ± sample SD was 0.942 ± 0.020 for Dice, 0.962 ± 0.016 for precision, 0.925 ± 0.050 for sensitivity, 0.996 ± 0.002 for specificity, and 0.940 ± 0.021 for clDice. Mean absolute connected-component error was 214 ± 122 components per validation volume, AVD was 6.032 ± 4.565%, and Kappa was 0.935 ± 0.022 (Table 2). Volume-level metric manifests are included with the results package to link each measurement to its reconstructed source stack.

#### 4.1.2 Comparison with 2D and 3D architectures

Below we compare DeepBranchAI against 2D and 3D U-Net (Ronneberger et al., 2015; Çiçek et al., 2016; Isensee et al., 2021) and 2D nnU-Net (Table 3). This is a Stage 3 segmentation-model comparison using the same expert-refined label source where available. It is not an annotation-strategy benchmark. For the reported metrics, each model was trained for 100 epochs using the same subject-rooted 5-fold validation groups defined at the original-volume level, with preprocessing, label generation, and expert refinement held fixed. Metrics were computed volume-wise on full 128-slice held-out volumes, with 2D predictions stitched into 3D volumes and 3D predictions assembled as full-volume masks before evaluation. Stage 1 and Stage 2 label generation were held fixed, so the comparison replaces the Stage 3 segmentation model rather than the label-generation strategy.

Volume-wise evaluation allows clDice and absolute connected-component error to be measured on assembled 3D predictions rather than isolated patches. Across the architecture-comparison rows, DeepBranchAI scored highest on Dice, specificity, and Kappa, while the 2D U-Net had the lowest Dice because it cannot use through-plane context. The 3D U-Net improved substantially, and 2D nnU-Net improved over standard 2D U-Net through automatic configuration, but both remained below DeepBranchAI on the primary overlap and agreement measures.

Because clDice is directly relevant to branching topology, we also trained a controlled five-fold comparison with the same subject-rooted validation groups, architecture, patch size, batch size, training length, and reference-label source as the standard CE + Dice nnU-Net. Across folds, adding clDice to the training objective had only marginal effects on most metrics. Mean Dice changed from 0.942 to 0.941 and mean clDice changed from 0.940 to 0.941. The clearest difference was lower mean absolute connected-component error, from 214 to 183, while the remaining metrics changed only slightly (Table 3). We therefore retained the CE + Dice model as the primary released mitochondrial checkpoint and report clDice as an evaluation metric. A representative DeepBranchAI segmentation compared with its reference label is shown in Figure 4.

**Figure 4:**
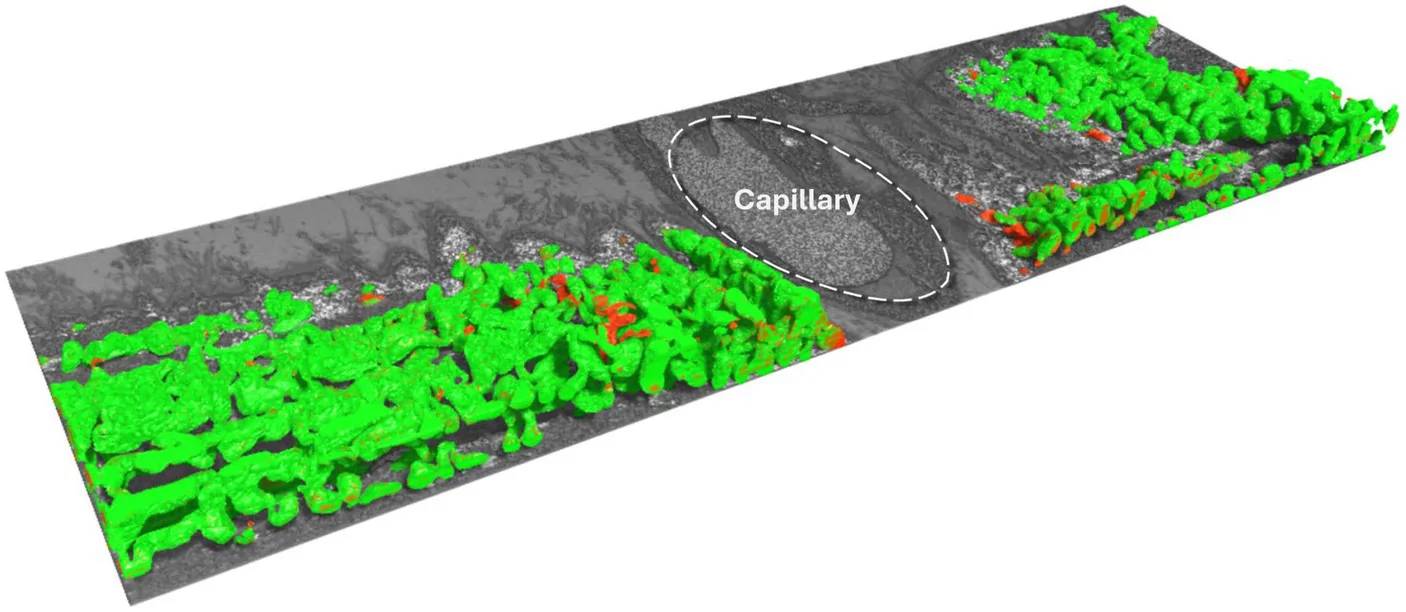
DeepBranchAI segmentation performance. Representative 3D pixel-to-pixel comparison between DeepBranchAI segmentation (green) and reference label (red) showing high overlap and discrepancies primarily contained in highly dense regions near the capillary.

#### 4.1.3 Reduced *Z*-context ablation

We also tested whether reducing the axial context from the released 352 × 352 × 128 patch size to 352 × 352 × 64 or 352 × 352 × 32 affected segmentation performance. Across five folds, the 128-slice setting had the highest mean Dice, precision, sensitivity, specificity, clDice, and Kappa and the lowest mean AVD. The 64-slice setting had the lowest mean absolute connected-component error, 203 compared with 214 for the 128-slice setting, whereas the 32-slice model had the lowest mean sensitivity and the highest mean absolute connected-component error. Overall, the 128-slice setting provided the strongest balance across the reported metrics and was retained as the final configuration (Table 4). Table 4 reports the unweighted mean ± sample SD across folds 0–4.

### 4.2 External transfer validation

External transfer was evaluated on paired image/reference-label datasets: 3D-IRCADb venous-system segmentation (Soler et al., 2010), Plant CT root segmentation (Harwood, 2024), and AeroPath airway CT (Støverud et al., 2024). We evaluate DeepBranchAI as a pretrained initialization for fine-tuning and compare it with scratch nnU-Net for each dataset. Domain-relevant comparator rows were also included: VesselFM for 3D-IRCADb venous-system CT, a 3D U-Net root-segmentation baseline informed by prior root-system 3D U-Network, and an airway-specific, cross-modality MurineAirwaySegmentation checkpoint adapted to AeroPath airway CT (Wittmann et al., 2024; Selzner et al., 2023; Isensee et al., 2023).

#### 4.2.1 Transfer learning strategy

External transfer experiments used the same paired image/reference-label folds for scratch and DeepBranchAI-pretrained fine-tuning within each dataset. 3D-IRCADb included 12 venous-system CT cases split across five folds, Plant CT included 29 root volumes split across five folds, and AeroPath included 27 airway CT volumes split across five folds. Target labels were the reference labels provided with each dataset or derived from the dataset. All nnU-Net transfer experiments used nnU-Net v2 preprocessing and planning, including dataset-specific resampling, normalization, patch-size selection, default augmentation, CE + Dice loss, and the nnUNetTrainer 100 epochs schedule. The nnU-Net experiments used the default nnU-Net v2 training configuration, including stochastic gradient descent with Nesterov momentum, momentum of 0.99, weight decay of 3 × 10^−5^, and polynomial learning-rate decay from an initial learning rate of 0.01.

Scratch models were trained from random initialization, while DeepBranchAI-pretrained models were initialized from the DeepBranchAI fold-0 mitochondrial checkpoint and all weights were allowed to update during fine-tuning. Comparator rows were generated by us using the same target-dataset folds and evaluated volume-wise with the same metric implementation. VesselFM and MurineAirwaySegmentation were initialized from public checkpoints and fine-tuned on the corresponding target data, while the 3D U-Net root baseline was trained from random initialization using the same Plant CT fold definitions.

VesselFM was fully fine-tuned for 10,000 optimizer steps, with all 31.4 million parameters updated. It used DiceCE loss weighted 0.9 for Dice and 0.1 for cross-entropy, AdamW with an initial learning rate of 1 × 10^−5^ and weight decay of 0.1, and cosine-annealing learning-rate decay to 1 × 10^−7^. The 3D U-Net root baseline was trained for 100 epochs with all weights updated. It used BCE-with-logits plus soft Dice loss, AdamW with an initial learning rate of 0.001 and weight decay of 1 × 10^−5^, and a constant learning rate. MurineAirwaySegmentation was initialized from the public Task145 fold-0 checkpoint and fully fine-tuned for 100 epochs on each AeroPath fold. It used Dice plus cross-entropy loss, stochastic gradient descent with Nesterov momentum, an initial learning rate of 0.01, momentum of 0.99, weight decay of 3 × 10^−5^, and polynomial learning-rate decay. VesselFM retained its native step-based training budget and was therefore not strictly epoch-matched to the 100-epoch models.

For each external dataset, nnU-Net was initialized either from scratch or from the DeepBranchAI fold-0 mitochondrial checkpoint and then fine-tuned on the target training folds. Metrics were computed against the provided reference labels. This design tests whether mitochondrial pretraining gives a useful initialization for other 3D branching-network tasks rather than assessing zero-shot generalization.

#### 4.2.2 Validation results

DeepBranchAI-pretrained fine-tuning had higher mean clDice than scratch nnU-Net in all three external datasets (Table 5). Paired out-of-fold analysis showed that the clDice difference was statistically significant in 3D-IRCADb venous CT after Holm correction (*p* = 0.010) and in AeroPath airway CT (*p* < 0.001), but not in Plant CT roots (*p* = 0.309). The Dice difference was significant in 3D-IRCADb (*p* = 0.004) and Plant CT roots (*p* = 0.038), but not in AeroPath (*p* = 0.672). Absolute connected-component error was significantly lower in AeroPath (*p* = 0.038). The differences in Plant CT roots (*p* = 0.309) and 3D-IRCADb (*p* = 0.791) were not significant. The adjusted *p* values and paired sample sizes are summarized in Table 6.

**Table 6:** Paired statistical comparison of DeepBranchAI-pretrained and scratch nnU-Net predictions.

Dataset	Paired volumes	Dice adjusted *p*	clDice adjusted *p*	Abs. CC error adjusted *p*
3D-IRCADb venous CT	12	0.004	0.010	0.791
Plant CT roots	29	0.038	0.309	0.309
AeroPath airway CT	27	0.672	<0.001	0.038

Values are Holm-adjusted two-sided Wilcoxon signed-rank test results calculated from held-out per-volume metrics.

Table 5 reports the unweighted mean ± sample standard deviation across folds 0–4. Standard deviations were calculated from the unrounded fold-level values, and percentage rows were calculated from the displayed mean values. For higher-is-better metrics, percentage difference was calculated as (DeepBranchAI − best non-DeepBranchAI comparator)/best comparator × 100. For absolute connected-component error, which is lower-is-better, the direction was reversed. Positive percentages therefore indicate a higher mean score or lower mean error for DeepBranchAI, depending on the metric. Inferential testing was restricted to the primary comparison between DeepBranchAI-pretrained and scratch nnU-Net because these conditions used the same architecture, target folds, and training procedure and differed only in model initialization. Figure 5 shows representative successful and challenging 3D-IRCADb transfer cases alongside scratch nnU-Net and VesselFM predictions. In panel A, the DeepBranchAI prediction is visibly more continuous than both comparators. Panel B is more challenging. Although the DeepBranchAI prediction is also visually more continuous, several branches remain disconnected or absent relative to the reference segmentation.

**Figure 5:**
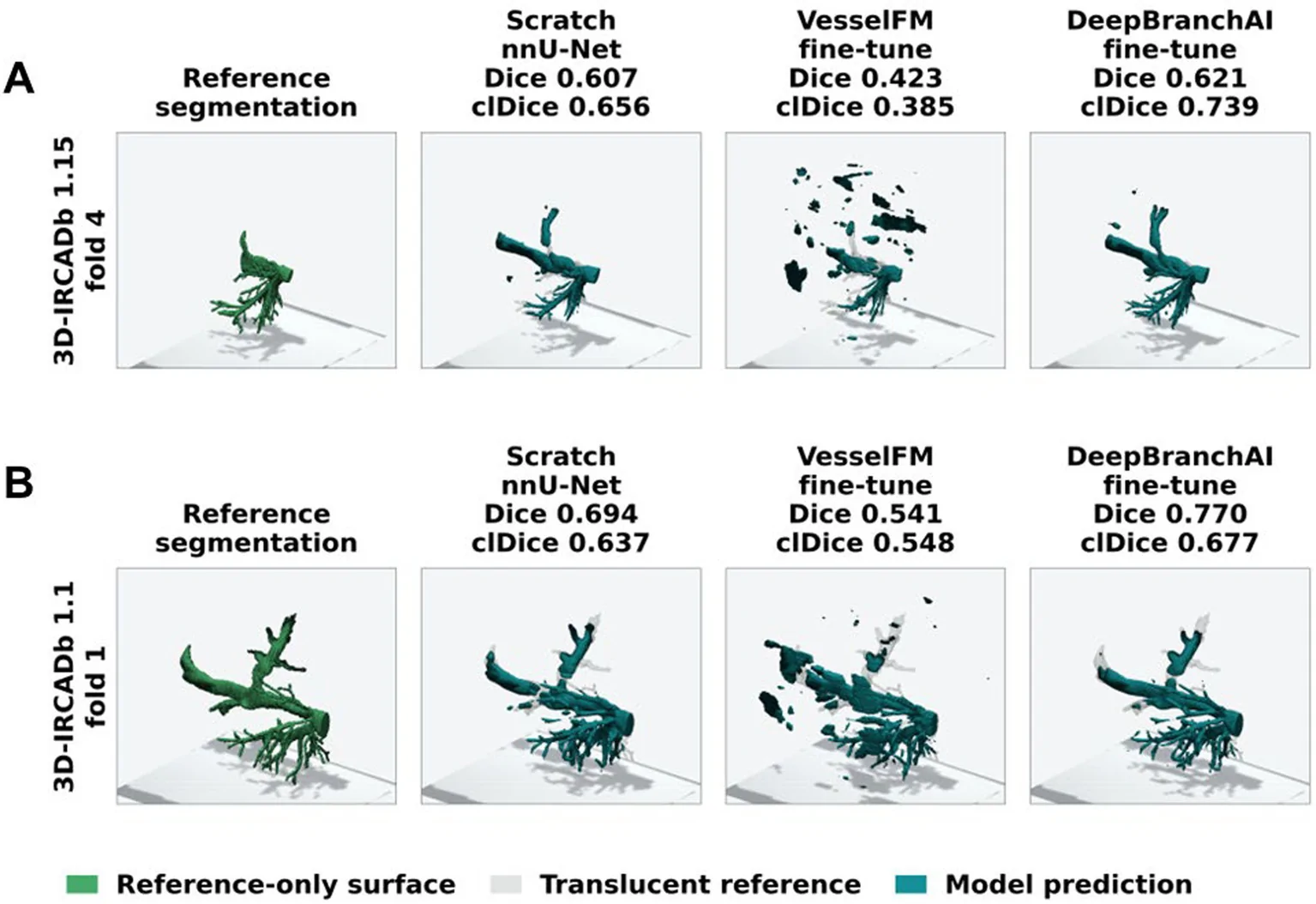
Qualitative external-transfer comparison on 3D-IRCADb venous CT. From left to right, columns show the reference segmentation, scratch nnU-Net prediction, VesselFM fine-tuned prediction, and DeepBranchAI fine-tuned prediction. Predictions are shown in teal with the reference segmentation overlaid in translucent gray. Views and display settings are matched within each row. **(A)** Successful case, 3D-IRCADb 1.15 (fold 4). **(B)** Challenging case, 3D-IRCADb 1.1 (fold 1). DeepBranchAI is visually more continuous than the comparator predictions in both cases.

## 5 Discussion

This study supports DeepBranchAI as a transferable 3D checkpoint for continuity-preserving segmentation of branching networks. DeepBranchAI was trained on expert-refined FIB-SEM labels of skeletal-muscle mitochondria and performed well in five-fold validation on the mitochondrial source task. This result shows that the model can learn the dense reticular architecture of skeletal-muscle mitochondria from volumetric reference labels (Glancy et al., 2015; Caffrey et al., 2019; Vincent et al., 2019). The broader test was whether the same checkpoint provided a useful pretrained initialization for external branching-network datasets. Across 3D-IRCADb venous CT, Plant CT roots, and AeroPath airway CT, DeepBranchAI-pretrained fine-tuning had higher mean clDice than scratch nnU-Net in all three datasets. Paired analysis supported the Dice and clDice differences in 3D-IRCADb, the Dice difference in Plant CT roots, and the clDice and absolute connected-component error differences in AeroPath. The remaining mean differences were not statistically significant after Holm correction.

The transfer results suggest that DeepBranchAI learned broader features than just mitochondrial image texture, including branch continuity and boundary discrimination. Skeletal-muscle FIB-SEM is a demanding setting for learning those broader features because mitochondria thread through a crowded cellular landscape that includes myofibrils, Z-disks, lipid droplets, nuclei, and other organelles (Glancy et al., 2015; Caffrey et al., 2019; Vincent et al., 2019). The model must separate a continuous reticular target from neighboring structures with similar local contrast, repeated boundaries, and thin interfaces between objects. This interpretation is consistent with recent volumetric electron-microscopy transfer-learning work showing that features learned from one organelle or tissue segmentation task can improve segmentation after fine-tuning on another (Xie et al., 2025). DeepBranchAI extends this idea across a larger modality and scale gap, where mitochondrial FIB-SEM pretraining remained useful after fine-tuning on CT roots, airways, and veins. DeepBranchAI-pretrained fine-tuning had higher mean clDice than scratch nnU-Net across all three external datasets and had lower mean absolute connected-component error in the plant-root and airway experiments.

The source dataset contained 20 volumes, while the 3D-IRCADb, Plant CT, and AeroPath datasets contained 12, 29, and 27 volumes, respectively. Five-fold cross-validation allowed every volume to contribute to held-out evaluation, but it does not replace validation in larger independent cohorts. The observed transfer performance may therefore depend on anatomical, acquisition, and pathological variation that is not fully represented in these datasets. Evaluation on larger and more diverse datasets would provide narrower uncertainty estimates and establish how consistently the learned representation transfers across populations and imaging settings.

Since the strongest transfer signal appeared in continuity-sensitive metrics, we also tested whether the mitochondrial source model should be trained directly with a topology-aware loss. In the five-fold loss-function comparison, adding clDice to the training objective had little effect on most metrics. Mean Dice changed from 0.942 to 0.941, while mean clDice changed from 0.940 to 0.941. Precision, sensitivity, specificity, AVD, and Kappa also differed only slightly. The clearest difference was in mean absolute connected-component error, which decreased from 214 to 183 components per validation volume. Prior work similarly treats topology-aware and overlap-based objectives as complementary. Shit et al. introduced clDice as a centerline-based score and soft loss for tubular segmentation but combined soft-clDice with soft-Dice rather than replacing volumetric overlap entirely (Shit et al., 2021). Acebes et al. noted that clDice loss can preserve topology while affecting segmentation accuracy and robustness to noisy annotations (Acebes et al., 2024). Shi et al. also described limitations in geometric detail and vessel-diameter balance (Shi et al., 2024). We therefore retained the standard CE + Dice nnU-Net as the released DeepBranchAI checkpoint and used clDice and absolute connected-component error as continuity-sensitive evaluation metrics.

These results also connect to a larger shift in biomedical segmentation. The field is moving from isolated task-specific models toward reusable 3D source models, out-of-the-box segmenters, and foundation checkpoints. nnU-Net made this shift practical by showing how much can be gained from standardizing preprocessing, architecture selection, training, and validation (Isensee et al., 2021). TotalSegmentator extended this logic into an out-of-the-box nnU-Net-trained CT segmentation model across many anatomical structures (Wasserthal et al., 2023). Models Genesis and Med3D made the related argument that 3D image structure itself can support reusable representations (Chen et al., 2019; Zhou et al., 2021). VesselFM pushed this idea in a specialized direction by training on heterogeneous vascular data to support vessel segmentation across modalities (Wittmann et al., 2024). This success aligns with findings by Tetteh et al., who showed that deep networks trained on tubular vessel structures can learn generalizable features rather than domain-specific textures (Tetteh et al., 2020). DeepBranchAI follows the same general direction, but from a different source domain. Instead of beginning with a broad medical-image corpus or a vessel-only corpus, it uses a structurally crowded mitochondrial FIB-SEM task as the pretraining problem. The use of nnU-Net keeps the checkpoint in a standardized framework that can be fine-tuned across the source task and the external CT datasets, while continuity is evaluated with clDice, connected-component error, the clDice-loss comparison, and external transfer performance. The results suggest that source-domain richness can matter even when source and target images are different.

Promptable foundation models such as MedSAM and SAM-Med3D provide complementary approaches to reusable medical segmentation (Ma et al., 2024; Wang et al., 2023). They were not included as Table 5 comparators because their prompt-based inference and adaptation procedures are not directly matched to the fully automated five-fold fine-tuning design evaluated here. A controlled comparison would require standardized prompts, adaptation rules, and training budgets across every target fold. The present comparison therefore focuses on scratch nnU-Net and pretrained checkpoints that can be adapted through fully automated target-domain fine-tuning.

## 6 Conclusion

DeepBranchAI provides a 3D nnU-Net checkpoint trained on expert-refined skeletal-muscle mitochondrial FIB-SEM labels for topology-sensitive segmentation of branching networks. Source-domain validation showed strong mitochondrial segmentation performance, and external fine-tuning had higher mean clDice than scratch nnU-Net in all three target datasets. Paired analysis supported differences in venous Dice and clDice, Plant CT Dice, and airway clDice and absolute connected-component error, while the remaining mean differences were not statistically significant after correction. These results support the use of structurally heterogeneous mitochondrial FIB-SEM data as a source domain for transferable 3D branching-network segmentation. Code, trained weights, preprocessing scripts, external-transfer scripts, validation scripts, and result tables are provided with the DeepBranchAI repository.

## 7 Future developments

Future work should move from single-source checkpoints toward shared 3D pretraining resources for branching-network segmentation. TotalSegmentator shows the value of organizing many CT anatomical labels into one reusable nnU-Net system (Wasserthal et al., 2023). MedSAM and SAM-Med3D show a related route, where large image-mask collections support reusable medical segmentation models (Ma et al., 2024; Wang et al., 2023). A branching-network equivalent would curate vascular, airway, root, mitochondrial, and porous-network volumes with explicit label definitions and continuity-sensitive metrics. Strong feature construction tailored to complex topologies may also be important for maximizing accuracy. Classical U-Net variants remain effective, but dense 3D volumes create heavy memory and parameter demands. Emerging quantum and quantum-inspired convolutional models offer one possible direction because resource-efficient QCNN layers and quantum 3D CNNs have been proposed as lower-resource ways to process structured image data (Song et al., 2024; Blekos & Kosmopoulos, 2021).

A second direction is topology-specific transfer learning beyond branching networks. DeepBranchAI tests this idea for continuous branching structures. Other structural classes raise different problems, such as loop-rich endoplasmic-reticulum networks and ER sheets (Lu et al., 2023), cardiac cavities and ring-like myocardium (Byrne et al., 2023), and porous materials where pore-space topology affects network extraction and physical measurements (Zubov et al., 2022; Wang et al., 2021). Checkpoints trained on dense examples of these structural classes could help models learn the relevant 3D geometry before fine-tuning on smaller target datasets.

## Data Availability

The datasets presented in this study can be found in online repositories. The names of the repository/repositories and accession number(s) can be found at: https://github.com/alexmaltsev/DeepBranchAI.

## Glossary

DSC: dice similarity coefficient
FIB-SEM: focused ion beam scanning electron microscopy
CNN: convolutional neural network
ML: machine learning
nnU-Net: no-new-U-net (self-configuring neural network framework)
RF: random forest
IoU: intersection over union
AVD: absolute volume difference
Key terms
Segmentation: The process of partitioning volumetric image data into regions corresponding to distinct structures or objects of interest.
Voxel: A three-dimensional pixel; the smallest distinguishable unit in volumetric imaging data.
Topological fragility: The vulnerability of branching networks where minor voxel misclassifications can break connectivity and fragment continuous structures.
Annotation: The process of manually labeling structures in images to create training data for machine learning models.
Reference-label generation: A multi-stage process in which conventional machine learning generates initial draft labels, which are then refined using deep learning and expert correction.
Ground truth: Expert-annotated reference data used as the correct answer for training and evaluating segmentation models.
Transfer learning: A technique where a model trained on one dataset or task is adapted to perform well on a different but related dataset or task.
Volumetric data: Three-dimensional image data composed of voxels (3D pixels) arranged in a regular grid.
Probability map: A grayscale image where each voxel’s intensity (0–1) represents the model’s confidence that the voxel belongs to the target structure.
Inference: The process of applying a trained model to new data to generate predictions or segmentations.
Patch size: The three-dimensional dimensions (in voxels) of image chunks processed by the neural network during training and inference.
Epoch: One complete pass through the entire training dataset during neural network training, after which model weights are updated based on cumulative learning.
Fine-tuning: The process of adapting a pre-trained neural network to a new task by continuing training on task-specific data.

## References

[ref1] Acebes C. Moustafa A. H. Camara O. Galdran A. (2024). The centerline-cross entropy loss for vessel-like structure segmentation: better topology consistency without sacrificing accuracy. In medical image computing and computer assisted intervention - MICCAI 2024. Lect. Notes Comput. Sci 15008, 710–720. doi: 10.1007/978-3-031-72111-3_67

[ref2] Arganda-Carreras I. Kaynig V. Rueden C. Eliceiri K. W. Schindelin J. Cardona A. . (2017). Trainable Weka segmentation: a machine learning tool for microscopy pixel classification. Bioinformatics 33, 2424–2426. doi: 10.1093/bioinformatics/btx180, 28369169

[ref3] Banavar J. R. Maritan A. Rinaldo A. (1999). Size and form in efficient transportation networks. Nature 399, 130–132. doi: 10.1038/20144, 10335841

[ref4] Blekos K. Kosmopoulos D. (2021). “A quantum 3D convolutional neural network with application in video classification,” in Advances in Visual Computing. ISVC 2021, eds. Bebis G. ., Lecture Notes in Computer Science, vol. 13017 (Cham: Springer), 601–612. doi: 10.1007/978-3-030-90439-5_47

[ref5] Breiman L. (2001). Random forests. Mach. Learn. 45, 5–32. doi: 10.1023/A:1010933404324

[ref6] Byrne N. Clough J. R. Valverde I. Montana G. King A. P. (2023). A persistent homology-based topological loss for CNN-based multiclass segmentation of CMR. IEEE Trans. Med. Imaging 42, 3–14. doi: 10.1109/TMI.2022.3203309, 36044487 PMC7614102

[ref7] Caffrey B. J. Maltsev A. V. Gonzalez-Freire M. Hartnell L. M. Ferrucci L. Subramaniam S. (2019). Semi-automated 3D segmentation of human skeletal muscle using focused ion beam-scanning electron microscopic images. J. Struct. Biol. 207, 1–11. doi: 10.1016/j.jsb.2019.03.008, 30914296 PMC6681459

[ref8] Cavicchioli M. Moglia A. Garret G. Sciarra A. Dubost F. Schnurre H. . (2025). D2-RD-UNet: a dual-stage dual-class framework with connectivity correction for hepatic vessels segmentation. Comput. Biol. Med. 195:110530. doi: 10.1016/j.compbiomed.2025.110530, 40580621

[ref9] Chatterjee S. Mattern H. Dörner M. Chatterjee S. Mattern H. Dörner M. . (2024). SMILE-UHURA challenge - small vessel segmentation at mesoscopic scale from ultra-high resolution 7T magnetic resonance angiograms. arXiv:2411.09593. doi: 10.48550/arXiv.2411.09593

[ref10] Chen S. Ma K. Zheng Y. (2019). Med3D: Transfer Learning for 3D Medical Image Analysis. arXiv:1904.00625. doi: 10.48550/arXiv.1904.00625

[ref11] Chen J. Mei J. Li X. Lu Y. Yu Q. Wei Q. . (2024). TransUNet: rethinking the U-net architecture design for medical image segmentation through the lens of transformers. Med. Image Anal. 97:103280. doi: 10.1016/j.media.2024.103280, 39096845

[ref12] Çiçek O. Abdulkadir A. Lienkamp S. S. Brox T. Ronneberger O. (2016). “3D U-net: learning dense volumetric segmentation from sparse annotation,” in Medical Image Computing and Computer-Assisted Intervention - MICCAI 2016, Lecture Notes in Computer Science, vol. 9901 (Cham: Springer), 424–432.

[ref13] Clough J. R. Byrne N. Oksuz I. Zimmer V. A. Schnabel J. A. King A. P. (2022). A topological loss function for deep-learning based image segmentation using persistent homology. IEEE Trans. Pattern Anal. Mach. Intell. 44, 8766–8778. doi: 10.1109/TPAMI.2020.3013679, 32886606 PMC9721526

[ref14] Diaz-Pinto A. Alle S. Nath V. Tang Y. Ihsani A. Asad M. . (2024). MONAI label: a framework for AI-assisted interactive labeling of 3D medical images. Med. Image Anal. 95:103207. doi: 10.1016/j.media.2024.103207, 38776843

[ref15] Funke J. Tschopp F. Grisaitis W. Sheridan A. Singh C. Saalfeld S. . (2019). Large scale image segmentation with structured loss based deep learning for connectome reconstruction. IEEE Trans. Pattern Anal. Mach. Intell. 41, 1669–1680. doi: 10.1109/TPAMI.2018.2835450, 29993708

[ref16] Glancy B. Hartnell L. M. Malide D. Yu Z. X. Combs C. A. Connelly P. S. . (2015). Mitochondrial reticulum for cellular energy distribution in muscle. Nature 523, 617–620. doi: 10.1038/nature14614, 26223627 PMC6988728

[ref17] Harwood R. (2024). Training data - plant root segmentation using 3D-Unet. Zenodo. doi: 10.5281/zenodo.14189395

[ref18] Isensee F. Jaeger P. F. Kohl S. A. A. Petersen J. Maier-Hein K. H. (2021). nnU-net: a self-configuring method for deep learning-based biomedical image segmentation. Nat. Methods 18, 203–211. doi: 10.1038/s41592-020-01008-z, 33288961

[ref19] Isensee F. Ziegler S. Trofimova D. (2023). nnU-net model weights for airway segmentation in murine lungs imaged with light sheet microscopy data. Zenodo. doi: 10.5281/zenodo.7892040

[ref20] Jiang W. Li Y. Jia Y. Feng Y. Yi Z. Chen M. . (2024). Ori-net: orientation-guided neural network for automated coronary arteries segmentation. Expert Syst. Appl. 238:121905. doi: 10.1016/j.eswa.2023.121905

[ref21] Jiang W. Li Y. Jia Y. Feng Y. Yi Z. Wang J. . (2024). Segmentation of coronary artery based on discriminative frequency learning and coronary-geometric refinement. Comput. Biol. Med. 181:109045. doi: 10.1016/j.compbiomed.2024.109045, 39180858

[ref22] Li R. Huang Y. J. Chen H. Liu X. Yu Y. Qian D. . (2022). 3D graph-connectivity constrained network for hepatic vessel segmentation. IEEE J. Biomed. Health Inform. 26, 1251–1262. doi: 10.1109/JBHI.2021.3118104, 34613925

[ref23] Litjens G. Kooi T. Bejnordi B. E. Setio A. A. A. Ciompi F. Ghafoorian M. . (2017). A survey on deep learning in medical image analysis. Med. Image Anal. 42, 60–88. doi: 10.1016/j.media.2017.07.005, 28778026

[ref24] Lu M. Christensen C. N. Weber J. M. Konno T. Läubli N. F. Scherer K. M. . (2023). ERnet: a tool for the semantic segmentation and quantitative analysis of endoplasmic reticulum topology. Nat. Methods 20, 569–579. doi: 10.1038/s41592-023-01815-0, 36997816

[ref25] Ma J. He Y. Li F. Han L. You C. Wang B. (2024). Segment anything in medical images. Nat. Commun. 15:654. doi: 10.1038/s41467-024-44824-z, 38253604 PMC10803759

[ref26] Marron J.S. Brain Artery Tree Data. University of North Carolina at Chapel Hill. Raw MRA Images Collected by CASILab at the University of North Carolina at Chapel Hill and Originally Distributed by the MIDAS Data Server at Kitware, Inc. https://marron.web.unc.edu/brain-artery-tree-data/ (Accessed July 7, 2026)

[ref27] Metzner R. Eggert A. van Dusschoten D. Pflugfelder D. Gerth S. Schurr U. . (2015). Direct comparison of MRI and X-ray CT technologies for 3D imaging of root systems in soil: potential and challenges for root trait quantification. Plant Methods 11:17. doi: 10.1186/s13007-015-0060-z, 25774207 PMC4359488

[ref28] Mou L. Zhao Y. Fu H. Liu Y. Cheng J. Zheng Y. . (2021). CS2-net: deep learning segmentation of curvilinear structures in medical imaging. Med. Image Anal. 67:101874. doi: 10.1016/j.media.2020.101874, 33166771

[ref29] Münch B. Trtik P. Marone F. Stampanoni M. (2009). Stripe and ring artifact removal with combined wavelet-Fourier filtering. Opt. Express 17, 8567–8591. doi: 10.1364/OE.17.008567, 19434191

[ref30] Nair M. Shepherd J. H. Best S. M. Cameron R. E. (2020). MicroCT analysis of connectivity in porous structures: optimizing data acquisition and analytical methods in the context of tissue engineering. J. R. Soc. Interface 17:20190833. doi: 10.1098/rsif.2019.0833, 32316883 PMC7211477

[ref31] Narotamo H. Silveira M. Franco C. A. (2024). 3DVascNet: an automated software for segmentation and quantification of mouse vascular networks in 3D. Arterioscler. Thromb. Vasc. Biol. 44, 1584–1600. doi: 10.1161/ATVBAHA.124.320672, 38779855 PMC11208061

[ref32] Pachitariu M. Stringer C. (2022). Cellpose 2.0: how to train your own model. Nat. Methods 19, 1634–1641. doi: 10.1038/s41592-022-01663-4, 36344832 PMC9718665

[ref33] Peddie C. J. Collinson L. M. (2014). Exploring the third dimension: volume electron microscopy comes of age. Micron 61, 9–19. doi: 10.1016/j.micron.2014.01.009, 24792442

[ref34] Ronneberger O. Fischer P. Brox T. (2015). “U-net: convolutional networks for biomedical image segmentation,” in Medical Image Computing and Computer-Assisted Intervention - MICCAI 2015, Lecture Notes in Computer Science, vol. 9351 (Cham: Springer), 234–241.

[ref35] Rudyanto R. D. Kerkstra S. van Rikxoort E. M. Fetita C. Brillet P. Y. Lefevre C. . (2014). Comparing algorithms for automated vessel segmentation in computed tomography scans of the lung: the VESSEL12 study. Med. Image Anal. 18, 1217–1232. doi: 10.1016/j.media.2014.07.003, 25113321 PMC5153359

[ref36] Selzner T. Horn J. Landl M. Pohlmeier A. Helmrich D. Huber K. . (2023). 3D U-net segmentation improves root system reconstruction from 3D MRI images in automated and manual virtual reality work flows. Plant Phenomics 5:0076. doi: 10.34133/plantphenomics.0076, 37519934 PMC10381537

[ref37] Shi P. Hu J. Yang Y. Gao Z. Liu W. Ma T. (2024). Centerline boundary dice loss for vascular segmentation. In medical image computing and computer assisted intervention - MICCAI 2024. Lect. Notes Comput. Sci 15008, 46–56. doi: 10.1007/978-3-031-72111-3_5

[ref38] Shit S. Paetzold J. C. Sekuboyina A. Ezhov I. Unger A. Zhylka A. . (2021). “clDice - a novel topology-preserving loss function for tubular structure segmentation,” in Proceedings of the IEEE/CVF Conference on Computer Vision and Pattern Recognition (CVPR 2021), 16560–16569. doi: 10.1109/CVPR46437.2021.01629

[ref39] Soler L. Hostettler A. Agnus V. Charnoz A. Fasquel J. B. Moreau J. . (2010). 3D Image Reconstruction for Comparison of Algorithm Database: a patient-specific Anatomical and Medical Image Database. Technical Report. Strasbourg, France: IRCAD.

[ref40] Song Y. Li J. Wu Y. Qin S. Wen Q. Gao F. (2024). A resource-efficient quantum convolutional neural network. Front. Phys. 12:1362690. doi: 10.3389/fphy.2024.1362690

[ref41] Støverud K.-H. Bouget D. Pedersen A. Leira H. O. Amundsen T. Langø T. . (2024). AeroPath: an airway segmentation benchmark dataset with challenging pathology and baseline method. PLoS One 19:e0311416. doi: 10.1371/journal.pone.0311416, 39356679 PMC11446458

[ref42] Subramaniam P. Kossen T. Ritter K. Hennemuth A. Hildebrand K. Hilbert A. . (2022). Generating 3D TOF-MRA volumes and segmentation labels using generative adversarial networks. Med. Image Anal. 78:102396. doi: 10.1016/j.media.2022.102396, 35231850

[ref43] Taha A. A. Hanbury A. (2015). Metrics for evaluating 3D medical image segmentation: analysis, selection, and tool. BMC Med. Imaging 15:29. doi: 10.1186/s12880-015-0068-x, 26263899 PMC4533825

[ref44] Tajbakhsh N. Shin J. Y. Gurudu S. R. Hurst R. T. Kendall C. B. Gotway M. B. . (2016). Convolutional neural networks for medical image analysis: full training or fine tuning? IEEE Trans. Med. Imaging 35, 1299–1312. doi: 10.1109/TMI.2016.2535302, 26978662

[ref45] Teikari P. Santos M. Poon C. Hynynen K. (2016). Deep learning convolutional networks for multiphoton microscopy vasculature segmentation. arXiv:1606.02382. doi: 10.48550/arXiv.1606.02382

[ref46] Tetteh G. Efremov V. Forkert N. D. Schneider M. Kirschke J. Weber B. . (2020). DeepVesselNet: vessel segmentation, Centerline prediction, and bifurcation detection in 3-D angiographic volumes. Front. Neurosci. 14:592352. doi: 10.3389/fnins.2020.592352, 33363452 PMC7753013

[ref47] Vincent A. E. White K. Davey T. Philips J. Ogden R. T. Lawless C. . (2019). Quantitative 3D mapping of the human skeletal muscle mitochondrial network. Cell Rep. 26, 996–1009.e4. doi: 10.1016/j.celrep.2019.01.010, 30655224 PMC6513570

[ref48] Wang H. Guo S. Ye J. Deng Z. Cheng J. Li T. . (2023). SAM-Med3D: towards general-purpose segmentation models for volumetric medical images. arXiv. doi: 10.48550/arXiv.2310.15161 40742874

[ref49] Wang S. Li C. Wang R. Liu Z. Wang M. Tan H. . (2021). Annotation-efficient deep learning for automatic medical image segmentation. Nat. Commun. 12:5915. doi: 10.1038/s41467-021-26216-9, 34625565 PMC8501087

[ref50] Wang Y. D. Shabaninejad M. Armstrong R. T. Mostaghimi P. (2021). Deep neural networks for improving physical accuracy of 2D and 3D multi-mineral segmentation of rock micro-CT images. Appl. Soft Comput. 104:107185. doi: 10.1016/j.asoc.2021.107185

[ref51] Wasserthal J. Breit H. C. Meyer M. T. Pradella M. Hinck D. Sauter A. W. . (2023). TotalSegmentator: robust segmentation of 104 anatomic structures in CT images. Radiology. Artif. Intell. 5:e230024. doi: 10.1148/ryai.230024, 37795137 PMC10546353

[ref52] Wei D. Lin Z. Franco-Barranco D. Wendt N. Liu X. Yin W. . (2020). MitoEM dataset: large-scale 3D mitochondria instance Segmentation from EM images. In Medical Image Computing and Computer Assisted Intervention - MICCAI 12265, 66–76. doi: 10.1007/978-3-030-59722-1_7, 33283212 PMC7713709

[ref53] West G. B. Brown J. H. Enquist B. J. (1997). A general model for the origin of allometric scaling laws in biology. Science 276, 122–126. doi: 10.1126/science.276.5309.122, 9082983

[ref54] Wittmann B. Wattenberg Y. Amiranashvili T. Shit S. Menze B. (2024). vesselFM: A Foundation Model for Universal 3D Blood Vessel Segmentation. arXiv:2411.17386. doi: 10.48550/arXiv.2411.17386

[ref55] Xie R. Mulcahy B. Darbandi A. Marwah S. Ali F. Lee Y. . (2025). Transfer learning improves performance in volumetric electron microscopy organelle segmentation across tissues. Bioinformatics. Advances 5:vbaf021. doi: 10.1093/bioadv/vbaf021, 40196751 PMC11974384

[ref56] Xu C. S. Hayworth K. J. Lu Z. Grob P. Hassan A. M. García-Cerdán J. G. . (2017). Enhanced FIB-SEM systems for large-volume 3D imaging. eLife 6:e25916. doi: 10.7554/eLife.25916, 28500755 PMC5476429

[ref57] Yang H. Wang W. Li C. Qi J. Wang P. Lei H. . (2022). Deep learning-based X-ray computed tomography image reconstruction and prediction of compression behavior of 3D printed lattice structures. Addit. Manuf. 54:102774. doi: 10.1016/j.addma.2022.102774

[ref58] Zhang M. Wu Y. Zhang H. Qin Y. Zheng H. Tang W. . (2023). Multi-site, multi-domain airway tree Modeling (ATM’22): a public benchmark for pulmonary airway segmentation. Med. Image Anal. 90:102957. doi: 10.1016/j.media.2023.102957, 37716199

[ref59] Zhou Z. Sodha V. Pang J. Gotway M. B. Liang J. (2021). Models Genesis. Med. Image Anal. 67:101840. doi: 10.1016/j.media.2020.101840, 33188996 PMC7726094

[ref60] Zubov A. S. Murygin D. A. Gerke K. M. (2022). Pore-network extraction using discrete Morse theory: preserving the topology of the pore space. Phys. Rev. E 106:055304. doi: 10.1103/PhysRevE.106.055304, 36559419

